# Solitary fibrous tumor of the greater omentum: case report and review of literature

**DOI:** 10.1186/s40792-021-01176-w

**Published:** 2021-04-15

**Authors:** Karim M. Eltawil, Carly Whalen, Bryce Knapp

**Affiliations:** 1 Department of Surgery, La Verendrye General Hospital, Riverside Health Care Facilities, 110 Victoria Avenue, Fort Frances, ON Canada; 2 Northern Ontario School of Medicine, Thunder Bay, ON Canada

**Keywords:** Solitary fibrous tumor, Omentum, Surgical resection

## Abstract

**Background:**

Solitary fibrous tumor (SFT) is a rare neoplasm of mesenchymal origin occurring most often in the visceral pleura, however, it has been described in almost every anatomic location of the human body. While most SFTs have a benign behavior, they can potentially be locally aggressive and demonstrate a malignant behavior.

**Case presentation:**

A 63 year-old male patient presented with lower abdominal pain and nausea and was noted on CT to have a large, heterogeneous lower abdominal mass with no evidence of metastatic disease. A surgical resection was performed and the mass appeared to be connected to the greater omentum with a vascular pedicle. It was not invading any intra-abdominal or pelvic organs. Pathology revealed an SFT of omental origin. The mitotic count was less than 4 per 10 high-power fields and all pathologic characteristics did not meet the criteria for a malignant SFT.

**Conclusions:**

We report an extremely rare case of SFT originating from the greater omentum. A multidisciplinary team approach was followed to plan the patient’s management strategy.

## Background

Solitary fibrous tumor (SFT) is a rare soft tissue tumor of mesenchymal origin that accounts for less than 2% of all soft tissue masses [1]. It was first described in the pleura in 1931 [2] and approximately 30% of SFTs arise in the thoracic cavity (pleura, mediastinum, lung parenchyma), while extra pleural tumors arise in the peritoneal cavity, retroperitoneal and pelvic soft tissue, abdominal viscera, deep soft tissue of extremities and head and neck [3]. The abdominal cavity represents the most frequent extra pleural site for SFTs; nevertheless, these tumors are much less common in the peritoneal than in the pleural cavity [4]. SFT involving the omentum have been previously reported in the literature. Herein, we report a rare case of a large SFT originating from the greater omentum and review the literature on the subject.

## Case presentation

**Fig. 1:**
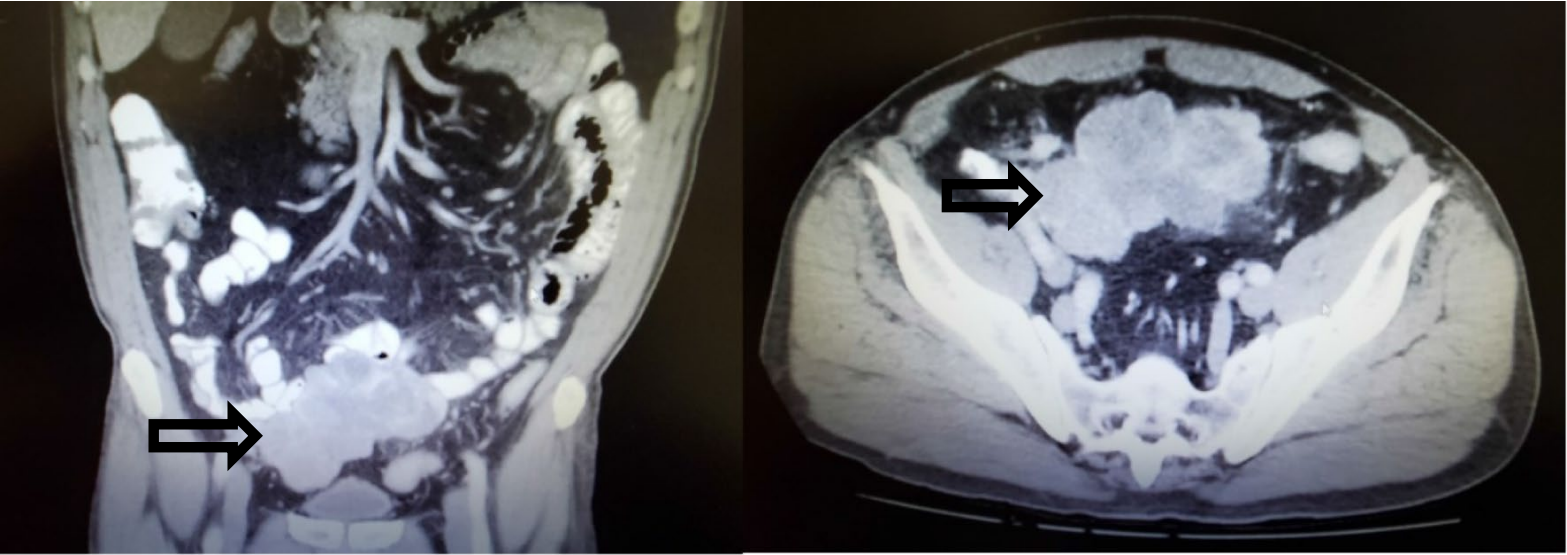
Heterogeneous SFT mass in the midline of the pelvis measuring 11.4 × 6.8 × 6.6 cm

A 63 year-old gentleman presented to the emergency department with non-specific periumbilical pain and nausea. He denied any vomiting, changes to his bowel habits, weight loss, fever or history of similar pain episodes. He was otherwise healthy and had no previous abdominal surgeries. On physical examination, there was some mild lower abdominal tenderness, but no mass was clinically palpable. A computed tomography (CT) of the abdomen and pelvis with oral and intravenous contrast was done and revealed a heterogeneous appearing mass like lesion in the midline of the pelvis measuring 11.4 × 6.8 × 6.6 cm. It showed peripheral enhancement with central hypodensity. The mass appears to be intimately associated with multiple segments of small bowel in the lower abdomen with no associated bowel obstruction (Fig. 1). The initial radiologist impression was suggestive of a possible gastrointestinal stromal tumor. A CT scan of the chest was performed as well and there was no evidence of metastatic disease in the abdominal or thoracic cavities.

The case was discussed in multidisciplinary tumor boards and the consensus was to proceed with surgical resection of the primary tumor. The patient was therefore taken to the operating room for a diagnostic laparoscopy, laparotomy and resection of the mass which was not found to involve the small bowel. During laparoscopic exploration, we did not identify any liver metastases or peritoneal deposits. The mass was occupying the middle portion of the lower abdominal cavity. We elected to convert to an open procedure due to concerns of disrupting the integrity of the tumor during laparoscopic dissection given its large size and weight. It was connected to the greater omentum with a vascular pedicle and slightly adherent to epiploic appendages of the sigmoid colon. An intraoperative flexible sigmoidoscopy was performed and was unremarkable. The mass was not invading any intra-abdominal structures and there was no evidence of metastatic disease. It was resected en bloc with adequate margin on the omental pedicle. The patient had an uneventful recovery and was discharged home on postoperative day three.

**Fig. 2:**
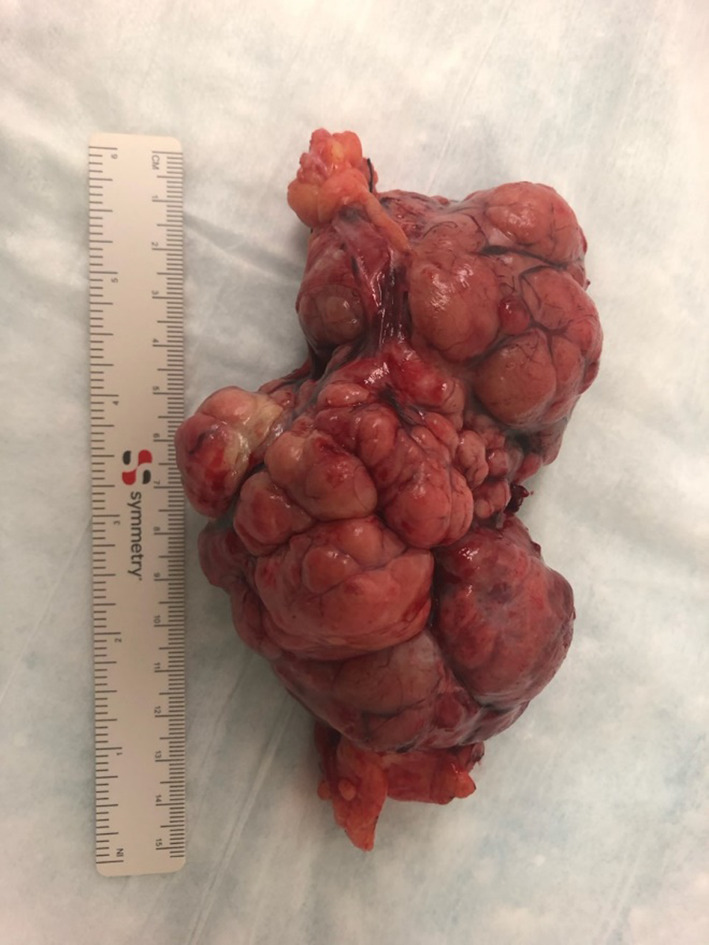
Tumor after complete surgical resection, encapsulated bosselated mass

**Fig. 3:**
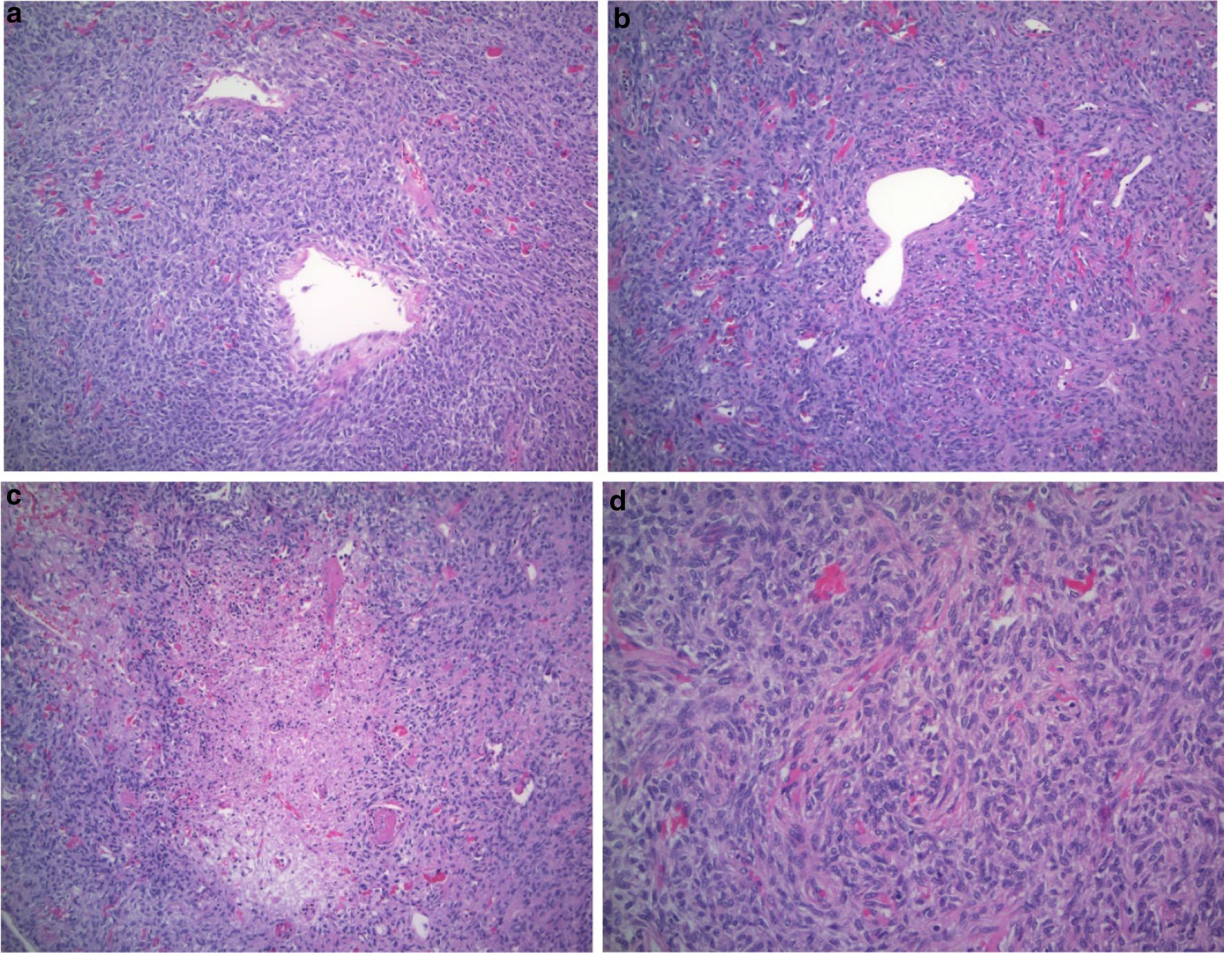
Hematoxylin and eosin-stained sections: **a**, **b** thin-walled vessels with staghorn-like appearance (+ 100 magnification power), **c** areas of necrosis (+ 100 magnification power), **d** spindle cells and collagen deposits (+ 200 magnification power)

**Fig. 4:**
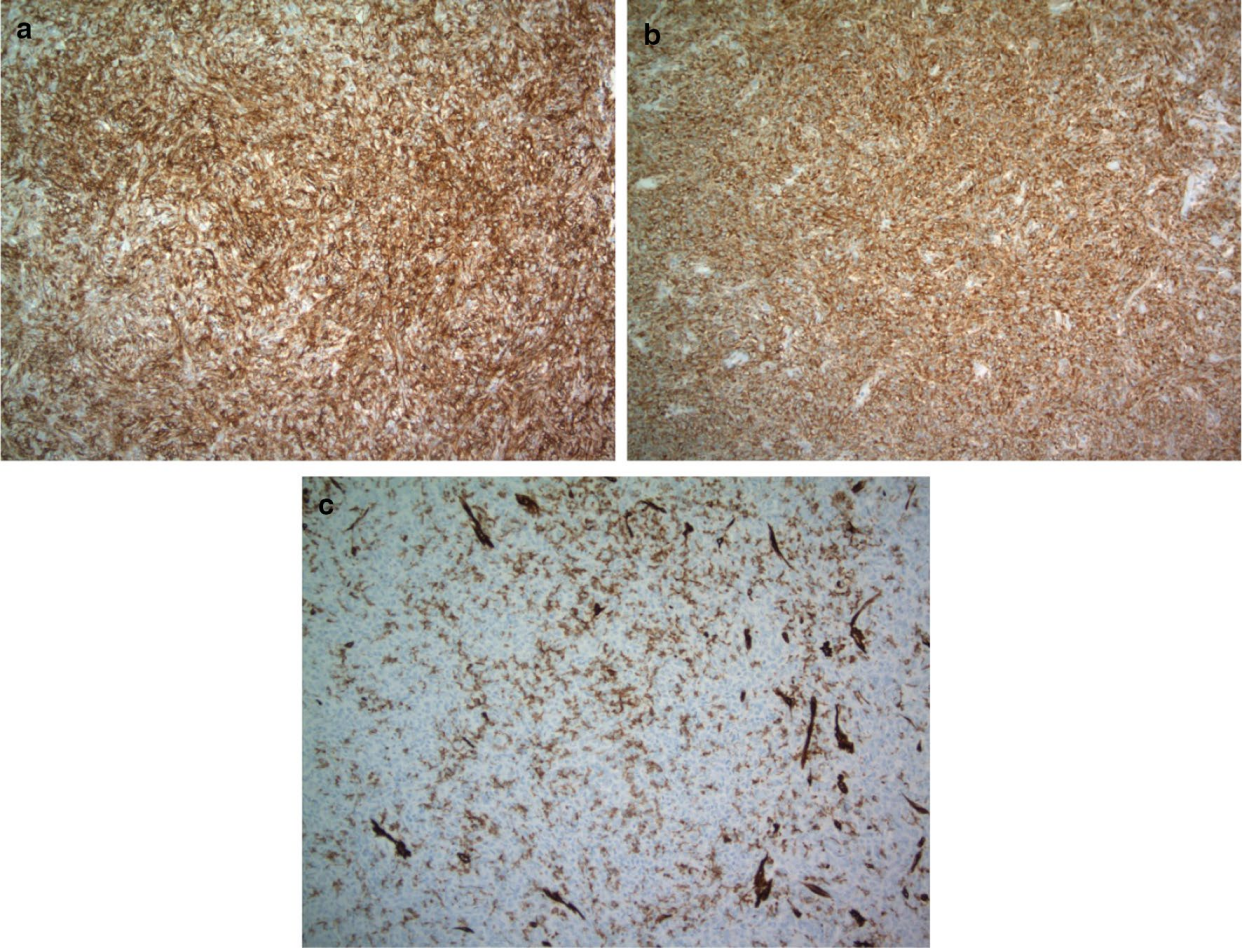
Immunohistochemical test (+ 100 magnification power): immunohistochemical test showing the tumor was positive for CD34 (**a**), and bcl-2 (**b**), and negative for CD31 (**c**)

Pathology revealed an SFT arising from the omentum. It was described as a finely encapsulated bosselated mass with a rubbery texture and weighed 225 g (Fig. 2). Microscopic examination showed a cellular lesion that was not invading the thin capsule with areas of necrosis, which likely represents infarction with a focal histiocytic reaction. The lesion was composed of plump spindled or slightly epithelioid cells in a small, storiform pattern. Nuclei appeared mildly atypical and relatively monomorphic. The mitotic count was less than 4 per 10 high-power fields and no abnormal mitoses were seen, so the lesion did not meet the criteria for a malignant SFT. However, given the fact that mitoses are not rare, it was best considered of unknown biological potential. The lesion was relatively vascular with abundant capillaries and background larger, thin-walled vessels (Fig. 3). The pedicle was identified arising from the omentum and showed no evidence of tumor invasion. Immune histochemical staining demonstrated the tumor cells are negative from CD31, ASMA, S100, AE1/AE3, calretinin, desmin and CD117. The tumor was positive for vimentin, BCL-2 and CD34 (Fig. 4). CD31 develops the vessels in the background. The very rare tumor cell was positive for epithelial membrane antigen (EMA).

Given the benign nature of the tumor histopathology and the completeness of the resection with negative margins, adjuvant chemo- or radiation therapy was deemed not indicated in multidisciplinary discussions. At 6 months follow-up, the patient remains asymptomatic with no clinical or radiologic evidence of recurrence or metastatic disease. The team agreed that routine surveillance may not be required and imaging and other investigations could be done as needed if the patient develops concerning symptoms or signs on examination.

## Discussion

SFTs are rare soft tissue neoplasms of mesenchymal origin occurring most often in the visceral pleura; nevertheless, they have been described in different anatomic locations [3]. They most commonly present in the fifth and sixth decade of life and appear to be equally distributed between men and women [5, 6]. Between 50 and 80% of thoracic SFT are asymptomatic masses while symptomatic tumors usually present with non-specific chest complaints such as chest pain, cough or dyspnea [7]. The abdominal cavity is the second most common location of SFT which has been noted in intraperitoneal, retroperitoneal and pelvic locations. Similar to intrathoracic SFT, they are generally asymptomatic until they reach a large enough size to cause mass effect on the intra-abdominal viscera [1, 8]. Non-specific abdominal symptoms include pain, vomiting and urinary symptoms. Uncommonly, patients may present with paraneoplastic syndrome, most commonly non-islet cell hypoglycemia. Paraneoplastic syndromes may occur in patients with SFT arising in all sites [9].

A definitive diagnosis of SFT requires histologic confirmation. Because many have non-specific symptoms or are asymptomatic, they are often identified incidentally on imaging studies [10]. On computed tomography (CT) and magnetic resonance imaging (MRI), they usually appear as well-circumscribed hypervascular lobulated soft tissue masses with areas of hemorrhage and necrosis particularly in large tumors [5].

Conventional immunohistochemical (IHC) markers of SFT include CD34, Bcl2, CD99, and vimentin in the absence of actin, desmin, S100 protein, or EMA. Nevertheless, these markers are not specific for SFT and may be inconsistently expressed [11, 12]. More recently, a strong nuclear expression of the C-terminal part of STAT6 (signal transducer and activator of transcription 6) has been shown to be a highly sensitive and specific marker for SFT, with a sensitivity and specificity of 98% and greater than 85%, respectively. However, STAT6 positivity alone may not be sufficient to distinguish some cases of SFT from its histologic mimic well-differentiated/dedifferentiated liposarcoma [8, 13, 14].

The management of SFT should be discussed in multidisciplinary tumor boards and a complete en bloc surgical resection represents the mainstay of therapy for all localized tumors [15]. Obtaining adequate negative margins has been shown to decrease the rate of local recurrence and improve overall survival [16]. In cases with positive surgical margins, particularly in higher-grade SFT, surgical re-resection should be considered [17]. The use of radiation therapy is best decided on a case-by-case basis in the context of a multidisciplinary discussion. For resected SFT with certain higher risk features such as positive surgical margins or high mitotic count, the use of adjuvant radiation may prevent local recurrences; however, an overall survival benefit has not been established [18, 19]. The role of adjuvant chemotherapy for resected SFT is unknown and the effectiveness of standard cytotoxic chemotherapeutic agents remains relatively poor [10]. Given the lack of data and the low incidence of SFTs, the benefit of adjuvant radiation and chemotherapy remains unknown. Additional clinical studies are needed to better characterize the molecular pathways involved with SFT in order to treat it more effectively.

The majority of SFTs have a benign behavior and do not recur locally or distally; nevertheless, 10–25% of pleural SFTs recur at 10 years [20–23]. Factors associated with poor prognosis include recurrent tumors, macro- or microscopically positive resection margins, tumor size > 10 cm, the presence of > 4 mitoses/10 HPF, increased nuclear pleomorphism, increased cellularity and presence of malignant component [1]. Solitary fibrous tumors originating from the greater omentum are extremely rare. To the best of our knowledge, only 15 [24–38] cases have reported in the literature up till December 2020 (Table 1).

**Table 1:** Reported cases of greater omentum SFT in the literature

Author	Year	Age	Gender	Presentation	Surgical procedure	Tumor size	Pathology	F/U (months)	Recurrence
Michiura et al. [24]	2016	36	Male	Abdominal mass	Laparotomy	9 cm	Benign	8	No
Archid et al. [25]	2016	24	Male	Abdominal pain	Laparotomy	8 cm	Benign	48	No
Urabe et al. [26]	2015	52	Male	Asymptomatic	Mini-laparotomy	5 cm	Benign	11	No
Osawa et al. [27]	2014	32	Female	vaginal bleeding	Laparoscopy	4.8 cm	Benign	12	No
Tarrga et al. [28]	2016	34	Female	Pelvic mass	Combined	6 cm	Malignant	32	No
Harada et al. [29]	2014	62	Female	Asymptomatic	Laparotomy	10 cm	Malignant	48	No
Moszynski [30]	2016	–	–	Pelvic mass	–	–	–	–	–
Cazejust et al. [31]	2015	68	Female	Abdominal pain	Laparoscopy	4.5 cm	Benign	–	–
Jung et al. [32]	2019	57	Male	Asymptomatic	Laparotomy	10 cm	Malignant	32	No
Garbin et al. [33]	2011	27	–	Asymptomatic	–	–	–	–	–
Sato et al. [34]	2014	85	Female	Abdominal mass	Laparotomy	19 cm	Benign	28	No
Patriti et al. [35]	2006	24	Male	Abdominal pain	Laparoscopy	3.2 cm	Benign	24	No
Zong et al. [36]	2012	29	Male	Abdominal mass	Laparotomy	21 cm	Benign	48	No

*F/U* follow-up

## Conclusions

We presented a case of SFT of the greater omentum that was discovered on a CT done to investigate vague abdominal symptoms. Surgical resection was performed with negative margins and the tumor did not meet the criteria of malignant SFTs, therefore the patient did not receive adjuvant therapy. A multidisciplinary team approach was followed to plan the management along its course of progression.

## Data Availability

Please contact author for data requests.

## Abbreviations

SFT: Solitary fibrous tumor
CT: Computed tomography
EMA: Epithelial membrane antigen
STAT6: Signal transducer and activator of transcription 6
IHC: Immunohistochemistry
HPF: High-power field

## References

[1] Gold JS, Antonescu CR, Hajdu C, Ferrone CR, Hussain M, Lewis JJ (2002). Clinicopathologic correlates of solitary fibrous tumors. Cancer.

[2] Klemperer P, Rabin CB (1931). Primary neoplasms of the pleura. A report of five cases. Arch Pathol.

[3] Miettinen M, Miettinen M (2010). Chapter 12: Solitary fibrous tumor, hemangiopericytoma, and related tumors. Modern soft tissue pathology: tumours and non-neoplastic conditions.

[4] Young RH, Clement PB, McCaughey WTE (1990). Solitary fibrous tumors (‘fibrous mesotheliomas’) of the peritoneum A report of three cases and a review of the literature. Arch Pathol Lab Med.

[5] Robinson LA (2006). Solitary fibrous tumor of the pleura. Cancer Control.

[6] Demicco EG, Park MS, Araujo DM, Fox PS, Bassett RL, Pollock RE (2012). Solitary fibrous tumor: a clinicopathological study of 110 cases and proposed risk assessment model. Mod Pathol.

[7] Sung SH, Chang JW, Kim J, Lee KS, Han J, Park SI (2005). Solitary fibrous tumors of the pleura: surgical outcome and clinical course. Ann Thorac Surg.

[8] Doyle LA, Vivero M, Fletcher CDM, Mertens F, Hornick JL (2014). Nuclear expression of STAT6 distinguishes solitary fibrous tumor from histologic mimics. Mod Pathol.

[9] Herrmann BL, Saller B, Kiess W, Morgenroth K, Drochner K, Schroder T (2000). Primary malignant histiocytoma of the lung: IGF-II producing tumor induces fasting hypoglycemia. Exp Clin Endocrinol Diabetes.

[10] Davanzo B, Emerson RE, Lisy M, Koniaris LG, Kays JK (2018). Solitary fibrous tumor. Transl Gastroenterol Hepatol.

[11] Brunnemann RB, Ro JY, Ordonez NG, Mooney J, El-Naggar AK, Ayala AG (1999). Extrapleural solitary fibrous tumors: a clinicopathologic study of 24 cases. Mod Pathol.

[12] Magro G, Bisceglia M, Michal M, Eusebi V (2002). Spindle cell lipoma-like tumor, solitary fibrous tumor and myofibroblastoma of the breast: a clinic-pathologic analysis of 13 cases in favor of underlying histogenic concept. Virchows Arch.

[13] Doyle LA, Tao D, Marino-Enriquez A (2014). STAT6 is amplified in a subset of dedifferentiated liposarcoma. Mod Pathol.

[14] Koelsche C, Schweizer L, Renner M, Warth A, Jones DT, Sahm F (2014). Nuclear relocation of STAT6 reliably predicts NAB2-STAT6 fusion for the diagnosis of solitary fibrous tumor. Histopathology.

[15] Cardillo G, Lococo F, Carleo F, Martelli M (2012). Solitary fibrous tumors of the pleura. Curr Opin Pulm Med.

[16] Kayani B, Sharma A, Sewell MD, Platinum J, Olivier A, Briggs TW (2018). A review of the surgical management of extrathoracic solitary fibrous tumors. Am J Clin Oncol.

[17] - National Comprehensive Cancer Network. Soft Tissue Sarcoma (V 2.2018). https://www.nccn.org/professionals/physician/gls/pds/sarcoma.pdf. Accessed 18 Oct 2018.

[18] Gholami S, Cassidy MR, Kirane A, Kuk D, Zanchelli B, Antonescu CR (2017). Size and location are the most important risk factors for malignant behavior in resected solitary fibrous tumors. Ann Surg Oncol.

[19] Wilky BA, Montgomery EA, Guzzetta AA, Ahuja N, Meyer CF (2013). Extrathoracic location and "borderline" histology are associated with recurrence of solitary fibrous tumors after surgical resection. Ann Surg Oncol.

[20] England DM, Hochholzer L, McCarthy MJ (1989). Localized benign and malignant fibrous tumors of the pleura. A clinicopathologic review of 223 cases. Am J Surg Pathol..

[21] de Perrot M, Fischer S, Bründler MA, Sekine Y, Keshavjee S (2002). Solitary fibrous tumors of the pleura. Ann Thorac Surg.

[22] Schirosi L, Lantuejoul S, Cavazza A, Murer B, Yves Brichon P, Migaldi M (2008). Pleuro-pulmonary solitary fibrous tumors: a clinicopathologic, immunohistochemical, and molecular study of 88 cases confirming the prognostic value of de Perrot staging system and p53 expression, and evaluating the role of c-kit, BRAF, PDGFRs (alpha/beta), c-met, and EGFR. Am J Surg Pathol.

[23] Salas S, Resseguier N, Blay JY, Le Cesne A, Italiano A, Chevreau C (2017). Prediction of local and metastatic recurrence in solitary fibrous tumor: construction of a risk calculator in a multicenter cohort from the French Sarcoma Group (FSG) database. Ann Oncol.

[24] Michiura T, Yamabe K, Hayashi N, Miyazaki Y, Sugimoto S, Kojima K (2016). A surgical case of solitary fibrous tumor originating from the greater omentum. Gan To Kagaku Ryoho.

[25] Archid R, Schneider CC, Adam P, Othman A, Zieker D, Königsrainer A (2016). Hemangiopericytoma/solitary fibrous tumor of the greater omentum: a case report and review of the literature. Int J Surg Case Rep.

[26] Urabe M, Yamagata Y, Aikou S, Mori K, Yamashita H, Nomura S (2015). Solitary fibrous tumor of the greater omentum, mimicking gastrointestinal stromal tumor of the small intestine: a case report. Int Surg..

[27] Osawa H, Nishimura J, Inoue A, Ueda M, Mokutani Y, Miyo M (2014). A case of solitary fibrous tumor from the greater omentum resected via laparoscopic surgery. Gan To Kagaku Ryoho.

[28] Rodriguez Tarrega E, Hidalgo Mora JJ, Paya Amate V, Vega OO (2016). Solitary fibrous tumor of the greater omentum mimicking an ovarian tumor in a young woman. Gynecol Oncol Rep.

[29] Harada N, Nobuhara I, Haruta N, Higashiura Y, Watanabe H, Ohno S (2014). Concurrent malignant solitary fibrous tumor arising from the omentum and grade 3 endometrial endometrioid adenocarcinoma of the uterus with p53 immunoreactivity. Case Rep Obstet Gynecol.

[30] Moszynski R, Szubert S, Tomczak D, Saad A, Samulak D, Sajdak S (2016). Solitary fibrous mass of the omentum mimicking an ovarian tumor: case report. Eur J Gynaecol Oncol.

[31] Cazejust J, Wendum D, Bourrier A, Chafai N, Menu Y (2015). Solitary fibrous tumor of the greater omentum. Diagn Interv Imaging.

[32] Jung CY, Bae JM (2019). Primary omental malignant solitary fibrous tumour, an extremely rare malignancy: a case report and review of the literature. Arab J Gastroenterol.

[33] Garbin O, Hummel M, Diana M, Wattiez A (2011). Solitary fibrous tumor of the greater omentum. J Minim Invasive Gynecol..

[34] Sato T, Yamaguchi S, Koyama I, Okada Y, Kato Y (2014). Acute life-threatening portal venous dilatation induced by a huge solitary fibrous tumor of the omentum. Hepatogastroenterology..

[35] Patriti A, Rondelli F, Gullà N, Donini A (2006). Laparoscopic treatment of a solitary fibrous tumor of the greater omentum presenting as spontaneous haemoperitoneum. Ann Ital Chir..

[36] Zong L, Chen P, Wang GY, Zhu QS (2012). Giant solitary fibrous tumor arising from greater omentum. World J Gastroenterol.

[37] Salem AM, Bateson PB, Madden MM (2008). Large solitary fibrous tumor arising from the omentum. Saudi Med J.

[38] Mosquera JM, Fletcher CD (2009). Expanding the spectrum of malignant progression in solitary fibrous tumors: a study of 8 cases with a discrete anaplastic component–is this dedifferentiated SFT?. Am J Surg Pathol.

